# Examining explainable clinical decision support systems with think aloud protocols

**DOI:** 10.1371/journal.pone.0291443

**Published:** 2023-09-14

**Authors:** Sabrina G. Anjara, Adrianna Janik, Amy Dunford-Stenger, Kenneth Mc Kenzie, Ana Collazo-Lorduy, Maria Torrente, Luca Costabello, Mariano Provencio

**Affiliations:** 1 Accenture The Dock, Dublin, Ireland; 2 Accenture Labs Dublin, Dublin, Ireland; 3 Medical Oncology Department, Hospital Universitario Puerta de Hierro, Madrid, Spain; Deakin University, AUSTRALIA

## Abstract

Machine learning tools are increasingly used to improve the quality of care and the soundness of a treatment plan. Explainable AI (XAI) helps users in understanding the inner mechanisms of opaque machine learning models and is a driver of trust and adoption. Explanation methods for black-box models exist, but there is a lack of user studies on the interpretability of the provided explanations. We used a Think Aloud Protocol (TAP) to explore oncologists’ assessment of a lung cancer relapse prediction system with the aim of refining the purpose-built explanation model for better credibility and utility. Novel to this context, TAP is used as a neutral methodology to elicit experts’ thought processes and judgements of the AI system, without explicit prompts. TAP aims to elicit the factors which influenced clinicians’ perception of credibility and usefulness of the system. Ten oncologists took part in the study. We conducted a thematic analysis of their verbalized responses, generating five themes that help us to understand the context within which oncologists’ may (or may not) integrate an explainable AI system into their working day.

## Introduction

Machine learning-based tools are increasingly being implemented to improve the efficiency and safety of healthcare. Machine learning (ML) has made it increasingly possible to provide patient-specific recommendations by using individual patient data [1]. However, academic publications have also highlighted the little impact these tools have had on the delivery of evidence-based medicine, a reflection of poor acceptance and adoption [2–4]. It is imperative that ML developers understand the perspectives of end-users by considering the specific use contexts to mitigate adoption issues later. This study describes the use of the Think Aloud Protocol (TAP) [5] to examine clinician needs, with the aim of integrating user feedback in the development of an explainable AI model fo predicting lung cancer recurrence risk.

Black-box ML, where the inner workings of the system that support the outputs are awkward to explain, have led to problems of non-adoption [6]. Five challenges of ML adoption for healthcare providers recur: (i) the AI learning curve; (ii) the cognitive burden of another source of information; (iii) the opportunity costs of using ML output *versus* seeing a patient in person; (iv) alert fatigue, and (v) concerns over bias [7]. Steps could be taken to bridge the gap in understanding, not least by explaining the decisions of ML systems. In response to black-box ML, the field of explainable AI emerged with the aim of “providing explanations catered to human understanding, trust, and transparency” [8].

There is an increasing legal need for AI systems to be explainable [9]. In April 2021, the European Commission proposed the first ever legal framework to regulate AI. When this regulation is enacted, high-risk AI such as the one used in clinical decisions should “facilitate the interpretation of the outputs of AI systems by the users” (Article 13) [2] safeguarding users’ rights to ‘meaningful information about the logic involved’ in automated decisions [14]. The Institute of Electrical and Electronics Engineer (IEEE) additionally is developing a standard for XAI that “defines mandatory and optional requirements and constraints that need to be satisfied for an AI method, algorithm, application or system to be recognized as explainable” [1]. While there is a long road ahead of the standard becoming approved and thereafter, implemented, these changes point to regulators and lawmakers becoming aware of the importance of AI systems to be interpretable.

Explainable AI lays the foundations in demystifying and understanding the inner mechanisms of ML models and is a fundamental requirement for a trustworthy AI system. Explanations for AI systems are interpretable descriptions of the system behavior, created with the goal of clarifying how automated decisions have been made. They come in many shapes, and their design rationale and use cases vary considerably [10]. There are two main branches of how to ensure model explainability in the AI system, one is by developing a model that is interpretable by design [11] e.g., logistic regression, decision trees, among others, while another approach is to apply post-hoc explanation techniques on top of the prediction obtained from an opaque, black-box model [12]. The post-hoc explanation approaches vary significantly among themselves as the notion of explanation is not agreed upon in the literature [13, 14]. We can have explanations as unquantifiable as visualizations of a model’s internal states, or as quantifiable as feature relevance methods. In this work, the space of “explanations” is narrowed down to local explanations—meaning that the single prediction (risk of relapse score) is accompanied by an object called “explanation”, which is linked to the prediction in such a way that it attempts to explain the score to the user of the model. This task has been approached differently, e.g., via analyzing the gradients derived through training the model and quantifying the impact of training instances on the output predictions, other approaches involve training a simplified model from the original black-box model to provide a model that is transparent and therefore could be interpreted more easily. Other approaches focus on providing the most similar training examples for the predicted one–these we call example-based explanations [14–16]. The list is not exhaustive, and we recommend recalling reviews such as [12].

Explanations greatly help make sense of automated predictions in high-stakes scenarios [4], that is, tasks with significant, life-changing consequences where the nature of the problem is hard to formalize in mathematical terms, and where ethical questions arise (for example, in automated underwriting, or in clinical decision-support systems (CDSS)). It is arguable that explainable AI has become essential in healthcare where decisions always must be accountable and anchored in a broader biomedical context. It has been noted that effective integration of CDSS with clinical workflow is only possible when clinicians’ needs are anticipated [17].

This work was done within the scope of the CLARIFY (Cancer Long Survivor Artificial Intelligence Follow-up) project. CLARIFY (Cancer Long Survivor Artificial Intelligence Follow-up) is a 3.5-year project which aims to identify risk factors of patient deterioration following oncological treatment for lung cancer, breast cancer, and lymphoma. It is a European Union-funded Horizon 2020 consortium which is a partnership of 12 institutions from five countries (Spain, Germany, Portugal, Ireland, and the United Kingdom working together throughout the project framework. The leading clinical partner is the Puerta de Hierro Hospital in Madrid, Spain.

The ML development is described in [31] (accepted in the Journal of Clinical Oncology Clinical Cancer Informatics). The afore-mentioned article describes in detail the training procedure and quantitative evaluation of the models’ performance along with data analysis aspects. The comparison between graph machine learning models and conventional tabular models is made. Two explanations approaches are described there as well–one example-based explanations for graph machine learning (novel approach built for the project) and one SHAP (SHapley Additive exPlanations) for tabular models. Both systems were presented to the clinicians in the final phase of the project (not described in this work) and were implemented in the research tool accessible to the clinicians at the hospital. The explanation method for graph machine learning, being novel, required additional intrpretability testing, following the evaluation phases of explainable approaches proposed by Doshi-Velez and Kim [18].

While attempts have been made to make AI outputs explainable, there remains a question of how users who need to incorporate ML models into their decision making assess the quality of ML outputs and explanations. We hypothesize that clinicians’ assessment of the quality of an ML prediction and explanation system is moderated by their perception of how *credible* and *useful* the system is: in the case of CDSS, this will likely be a direct result of the alignment between which clinical features they deem important and which variables were used by the model to reach its predictions, and how. To test this hypothesis in the context of the CLARIFY project, we used a Think Aloud Protocol (TAP): a method to elicit the desired information without priming the participants.

Developers must consider the specific context and who the intended users are of a CDSS when determining how to elicit meaningful information from them. In the context of CLARIFY, the vastly different academic backgrounds of AI engineers, expert users (oncologists), and social science researchers render semi-structured and structured interviews vulnerable to distortions linked to a lack of shared understanding. Eliciting user feedback to yield system improvements through the right method in a timely way is an enduring challenge in human-computer interaction research [19]. This study sets out to increase the fidelity and usability of user feedback to improve the next iteration of an explainable AI model.

There are a range of methods (some qualitative, some quantitative) to better understand user experience (UX). In general, these methods (i) concern themselves with the intuitive or unintuitive use of tangible manifestations of a system and (ii) tend to occur later in a system development cycle [20]. Our need though, is greater than understanding UX: it is a need to understand the orientation of an expert user (in this case, oncologists) to a system that purports to be useful to the exercise of clinical judgment (in this case, predicting relapse likelihood of patients with lung cancer).

Evidence shows that medical experts look for signals of credibility and scientific rigor when evaluating tools that are described as being complementary or assistive to their clinical judgment [21, 22]. It follows, then that we need to get a solid account of credibility and rigor as assessed by the clinicians when they first encounter a system that intends to help them, for example, in gauging the likelihood of relapse among their cancer patients.

While novel to the context of explainable AI, TAP has been used to analyze the process of clinical decision-making. Six publications (Table 1) [23–28] have detailed their use of TAP in qualitative and mixed method studies.

**Table 1:** Results of a literature search on PubMed, on the use of the think aloud protocol to assess clinical decision making.

Author	Year	*Title*	Phenomenon	Tool used	Type	Cited By
Thompson	2017	*A comparative study on the clinical decision-making processes of nurse practitioners vs. medical doctors using scenarios in a secondary care environment*	decision-making processes	think aloud + information processing theory	qualitative	7
Kilsdonk	2016	*Uncovering healthcare practitioners’ information processing using the think-aloud method: From paper-based guideline to clinical decision support system*	information processing while reviewing a guideline	think aloud	qualitative	23
Ericzén	2015	*Clinical Decision-Making in Community Children’s Mental Health: Using Innovative Methods to Compare Clinicians With and Without Training in Evidence-Based Treatment*	clinical decision-making	think aloud + naturalistic decision-making theory	qualitative	15
Nair	2015	*A Clinical Decision Support System for Chronic Pain Management in Primary Care: Usability testing and its relevance*	task performance using CDSS	think aloud	mixed	13
Press	2015	*Usability Testing of a Complex Clinical Decision Support Tool in the Emergency Department: Lessons Learned*	usability of a complex CDS tool in ED	think aloud + near-live simulations	qualitative	36
Li	2012	*Integrating usability testing and think-aloud protocol analysis with "near-live" clinical simulations in evaluating clinical decision support*	clinician interaction with CDS	think aloud + near-live simulations	qualitative	139

We kept Schwarz’s dictum in mind that getting as close as possible to ‘real time’ thinking of users/respondents is desirable [29]. Problems can arise when the research protocol discourages candor and accuracy, when questions are ambiguous and difficult to understand, or when the task exceeds research participants’ knowledge and limits of memory [29]. Lessons from a body of research around this topic suggests that self-report problems can be attenuated by asking questions in close temporal proximity to the event of interest, as doing so constrains the multiple meaning of questions, reduces memory and estimation problems, and facilitates access to episodic detail. We propose that the think aloud protocol (TAP) is a suitable tool to elicit a more faithful representation of what clinicians think, compared to structured or semi-structured interviews.

TAP refers to a type of data elicitation method also known as ‘concurrent verbalization,’ which means that subjects are asked to perform a task and to verbalize whatever crosses their mind during the task performance, such as upon exposure to ML prediction and explanation, and when assessing its helpfulness to achieve specific goals. It reduces the potential of interviewer bias. TAP has its origins in cognitive psychology, specifically in laboratory studies of human reasoning [30]. The theory underlying this method proposes that thoughts elicited by the method are a valid ‘sample’ of at least a subset of the thoughts involved in the mediation of the task being performed.

While related to classical introspection, in which a person analyses their own thought processes, TAP has been developed into a more rigorously controlled method of eliciting data on cognitive processes. After examining a large body of data, Ericsson and Simon argued that when elicited with care and appropriate instructions, TAP does not change the course or structure of thought processes, except for a slight slowing down of the process [30].

There are limitations to what kind of cognitive processes are accessible by TAP. Only information actively processed in the working memory can be verbalized, which means that unconscious processing is inaccessible. High cognitive load can hinder verbalization by using up all available cognitive resources. As a result, TAP has been considered useful in offering informative glimpses of cognitive processing in progress, but never a complete account. To form a more reliable picture, the TAP data can be complemented by examining the products of the process, i.e., a decision or assessment rating.

In this exploration, we use TAP supplemented by five binary response questions and two ecological validity questions. The binary response questions asked: 1) Was the AI system confusing or not confusing 2) Was the AI system overwhelming or not? 3) Was the AI system missing something or complete? 4) Was the AI system not useful or useful? and 5) Was the AI system misleading or clear? Ecological validity specifically examines if a given study’s variables and conclusions can be generalized into real-world situations, such as clinical practice [31, 32]. To the best of our knowledge, we are the first to use this modified Think Aloud protocol to assess clinicians’ response to a **novel explanation system** for a machine learning model.

## Methods

As part of the development of the lung cancer prediction and explanation system, we designed the current study. This study, embedded within CLARIFY, adopted an observational single-arm study design which aims to uncover clinicians’ assessment of the quality of a machine learning prediction and explanation system.

### Ethics

Central to the CLARIFY project’s design is the protection and dignity of European citizens, whose health data are transferred and processed by our system. The project’s technology was designed and developed with strict security and privacy measures. As CLARIFY deals with large amounts of data generated by patients and healthcare professionals, protocols have been put in place to ensure that all ethical concerns are addressed appropriately. This ensures that every aspect of the project complies with relevant national and EU legislations.

In particular, the data of the patients participating in the project was processed and handled according to Spain’s Organic law 3/2018, of December 5th, on personal data protection and guarantee of digital rights (LOPDGDD for the Spanish abbreviation). This law adopts the Regulation (EU) 2016/679 of the European Parliament and of the Council, of 27 April 2016, regarding the processing of personal data and on the free movement of such data (also known as the “GDPR”).

CLARIFY has established an Ethics Committee chaired by STELAR, one of the partners specializing in cybersecurity and data protection, who along with the project’s Data Protection Officer advises the consortium on how ethics-related matters should be handled within CLARIFY. Additionally, CLARIFY is assessed, approved, and monitored closely by the Clinical Research Ethics Committee of Hospital Universitario Puerta de Hierro.

This study does not interact with patients or patient data directly and asking end users about their assessment of the predictive and explanation model was considered an integral part of the ML system development. The consortium waived the requirement for a full ethical review after reviewing the study protocol (Appendix A in S1 File) and informed consent document. How we obtained informed consent from participants will be detailed in an upcoming section on Procedure.

### Machine learning use case

A CLARIFY sub-team developed predictive models based on integrated knowledge graphs, enabling intelligent, explainable decision support services to stratify oncology patients based on post-treatment complication risks. Specifically, this sub-team was assigned a task of predicting chances of patient’s relapse and of explaining such predictions.

Early in the development process, the CLARIFY project team held several ‘requirement gathering’ meetings and continued the gathering of desired features over emails and other asynchronous ways of communication (e.g., working on a common understanding of a database tables). This exercise involved all ML developers and two oncologists in the hospital, with the main purpose of compiling a list of features that the predictive and explanation system should incorporate. The resulting list from this asynchronous communication is recalled below in Table 2, as it originally appeared in [31].

**Table 2:** Full features list obtained including training features, identification, label, and features with missing values filtered out before training.

Features	Name		Description		Filter
**Features filtered with the selection criteria**					
(only values specified in the Filter column were included in the model)					
diagnosis_stage_t	Stage T		T stage of the patient’s tumor. Relates to the primary tumor’s size.		T1a, T1b, T1c, T2a, T2b, T0, Tx, Tis, T3
diagnosis_stage_n	Stage N		N stage of the patient’s tumor. Relates to the degree of spread to lymph nodes.		N0, N1
diagnosis_stage_m	Stage M		M stage of the patient’s tumor. Relates to the presence of metastasis.		M0
chemotherapy@t1_type	Type		Chemotherapy type conducted.		Adjuvant chemotherapy, QT intravenous, Adjuvant QT-RT
tumor_stage	Tumor Stage		Stage associated to the patient (I, II, III, IV‚. . .).		I, IA, IA1, IA2, IA3, IB, II, IIA, IIB, IIB
**Symptoms**					
symptom_asymptomatic	Asymptomatic		Whether or not the patient was asymptomatic.		-
symptom_cough	Cough		Whether or not the patient presented cough as symptom.		-
symptom_pain	Pain		Whether or not the patient presented pain as symptom.		-
symptom_dyspnea	Dyspnea		Whether or not the patient presented dyspnea as symptom.		-
symptom_hemoptits	Hemoptits		Whether or not the patient presented hemoptysis as symptom.		-
symptom_weight_loss	Weight Loss		Whether or not the patient presented weight loss as symptom.		-
symptom_anorexia	Anorexia		Whether or not the patient presented anorexia as symptom.		-
symptom_astenia	Astenia		Whether or not the patient presented asthenia as symptom.		-
symptom_others	Others		Whether or not the patient presented other symptoms.		-
**Comorbidities**					
comorbidity_alcoholism_or_ex_alcoholism	Alcoholism/Ex-Alcoholism		Name of the comorbidity associated to the patient.		-
comorbidity_asthma	Asthma				-
comorbidity_benign_prostatic_hyperplasia	Benign prostatic hyperplasia				-
comorbidity_cardiopathy	Cardiopathy				-
comorbidity_copd	COPD		Chronic obstructive pulmonary disease		-
comorbidity_depressive_syndrome_or_anxiety	Depressive syndrome/Anxiety		Name of the comorbidity associated to the patient.		-
comorbidity_diabetes_mellitus	Diabetes mellitus				-
comorbidity_dyslipidemia	Dyslipidemia				-
comorbidity_gastrointestinal	Gastrointestinal				-
comorbidity_hepatitis	Hepatitis				-
comorbidity_hta	HTA				-
comorbidity_hypercholesterolemia	Hypercholesterolemia				-
comorbidity_liver_disease	Liver disease				-
comorbidity_neurodegenerative_disorder	Neurodegenerative disorder				-
comorbidity_none	None		Name of the comorbidity associated to the patient.		-
comorbidity_obesity	Obesity				-
comorbidity_obstructive_sleep_apnea	Obstructive sleep apnea				-
comorbidity_others	Others				-
comorbidity_renal_disease	Renal Disease				-
comorbidity_tuberculosis	Tuberculosis				-
comorbidity_valscular_disease	Valscular disease				-
**Smoking Features**					
smoker	Smoker		Indicates the type of smoking status of the patient.		-
nb_cig_packs_year	Number of cigarettes packs/year		Number of cigarette packages consumed per year.		-
nb_cigs_day	Number of cigarettes/day		Number of cigarettes per day consumed by the patient.		-
lives_with_smokers	Lives with smoker		Whether or not the patient lives with smokers.		-
lived_with_smokers_20_years	Lived with smokers 20 years		Whether or not the patient has lived with smokers in the last 20 years.		-
**General Features**					
age	Age		Patient age at diagnosis, approximated with heuristic based on first available date for missing values.		-
race	Race		Race of the patient.		-
previous_cancer_type	Previous cancer type		Type of oncological antecedent experienced by the patient.		-
family_lung_cancer	Number of family members with lung cancer		Number of family relatives with lung cancer.		-
family_other_cancer	Number of family members with other cancer		Number of family relatives with other types of cancer.		-
gender	Gender		Gender of the patient.		-
**Diagnosis Features**					
histology_grade	Histology Grade		Histological degree presented by the patient’s tumor.		-
tumor_histology	Tumor Histology		Type of histology associated to the patient’s tumor.		-
diagnosis_ecog	ECOG		Value describing the patient’s performance status (in relationship with the scale indicated in the "scale" column).		-
diagnosis_synchronous_tumours	Synchronous tumors		Whether or not the patient has synchronous tumors.		-
adeno_histology	Histology subtype: adeno histology		-		-
histology_subtype	Histology Subtype		-		-
biomarker@diagnosis_ALK_IHQ	ALK IHQ		IHQ result of the ALK study.		-
biomarker@diagnosis_PD-L1	PD-L1		PDL1 result.		-
biomarker@diagnosis_type	Biomarker type		Marker studied.		-
biomarker@diagnosis_EGFR_negative	EGFR negative		Whether or not the result of this biomarker’s study was negative.		-
**Radiotherapy Features**					
radiotherapy@t1_type	Type		Type of radiotherapy conducted.		-
radiotherapy@t1_area	Area		Area radiated during the radiotherapy treatment.		-
radiotherapy@t1_dose	Dose		Dosage of radiotherapy applied to the patient (measured in Gray units).		-
radiotherapy@t1_fractioning	Fractioning		Number describing the amount of parts in which the radiotherapy dose was divided.		-
radiotherapy@t1_duration_days	Duration [days]		Days elapsed between start of radiotherapy and end of radiotherapy.		-
**Chemotherapy Features**					
chemotherapy@t1_start_time	Start Time [months]		Chemotherapy starts in months since the first recorded event in patient history.		-
chemotherapy@t1_regimen	Regimen		Class of chemotherapy process conducted on the patient.		-
**Surgery Features**					
surgery@t1_procedure1	Procedure 1		1st surgery iteration in which the surgery was conducted.		-
surgery@t1_time	Time		Surgery time in months since the first recorded event in patient history.		-
surgery@t1_type	Type		Type of surgery perfomed on the patient.		-
surgery@t1_resection_grade	Resection Grade		Degree of resection associated to the surgery,		-
surgery@t1_t_stage	Stage T		T stage of the patient. Measured before the conduction of surgery.		-
surgery@t1_n_stage	Stage N		N stage of the patient. Measured before the conduction of surgery.		-
surgery@t1_perioperative_death	Perioperative Death		Whether or not perioperative death took place within 30 days after surgery.		-
**Features with all values missing for the selected cohort (NOT included in the model)**					
mesothelioma		Histology subtype: mesothelioma		-	
small_cell_carcinom		Histology subtype: small cell carcinom			
carcinom		Histology subtype: carcinom			
surgery@t1_pathological_response		Pathological response		Type of pathological response to the surgery experienced by the patient.	
**Anonymized Identification and Labelling**					
relapse?		Relapse		Whether the patient relapsed or progressed. Used as a label.	
id		Id		Electronic Health Record. Anonymized unique identifier of a patient (excluded for tabular models, used in KGE models to represent patient nodes, needed by model design).	

The CLARIFY system relies on heterogenous data, starting from clinical patient data through various medical background knowledge, which can be represented effectively by knowledge graphs. The format of data that blends various sources is complex and more challenging than the single-source tabular data. Both predictive models, built on knowledge graphs, and many tabular machine learning models are not directly interpretable. To explain their predictions, one needs to apply post-hoc methods that interrogate the inner state of the model and recall relevant constructs. The difference is that for tabular models, being more popular and established, there are many more explainability methods developed than for relational learning models on graphs. It is essential to design explainability methods and protocols for evaluating them in projects like CLARIFY since predictive tools rely on heterogenous data represented as knowledge graphs. The explainable layer augmenting the predictive models is an example-based heuristic, called ExamplE, purpose-built for CLARIFY.

ExamplE is a post-hoc, local explanation subsystem that explains predictions of links returned by any knowledge graph embedding model architecture. It consists of four steps: sampling, filtering for examples, aggregating for prototype, and assembling the explanation graph. Details of this approach can be found in Janik and Costabello’s paper [33].

In this study, we utilized predictions and explanations provided by ExamplE to elicit medical experts’ thought processes and judgements of the AI system.

### Participants

Twenty (20) oncologists with at least 5 years of specialization training post-graduation from medical school and who would be end-users of the system were invited to participate in the study. These were medical doctors who have either attained full specialist status or are in the training program to attain full specialist status. Participants were required to have had the experience of using the hospital’s electronic health record system in which the CDSS is going to be embedded. This was taken as a criterion for their understanding of the variables stated in the explanation.

Eleven oncologists agreed to participate (response rate of 55%). One participant (Participant 8) was eventually removed from the analysis due to their involvement in the development of the predictive model. Responses from 10 participants (7 females, 3 males; age ranging from 35–45) remained in the analysis.

### Procedure

Oncologists were approached through a gatekeeper, a computer scientist who is part of the CLARIFY project team. The gatekeeper distributed the information leaflet pertaining to the study via email to all oncologists in the hospital. Oncologists independently responded to the gatekeeper via email indicating their willingness to participate in the study. Upon receipt of this email, the gatekeeper organized suitable dates and times for the video calls to take place. The gatekeeper then sent on a personalized video call link, a copy information leaflet, and informed consent form to each participant through Microsoft Outlook. Participants were informed that by accepting the video call invitation, they were thereby consenting to participating in the study. This acceptance is recorded in the Microsoft Outlook platform. At the start of the video calls, verbal consent was again sought. The gatekeeper was not part of the video calls.

We used Ericsson and Simon’s procedures for eliciting concurrent thoughts [30]. Researchers provided the participants with standardized instructions to concurrently think aloud. These included (a) informing the participant that they would be asked to look at two different stimuli in the form of a display interface, and (b) asking the participant to verbalize everything that they say to themselves as they would engage with the stimuli in a specific scenario. If participants were silent for any length of time during the tasks, they were reminded to keep talking aloud.

Video calls took place in Microsoft Teams platform. Only the call audio was recorded. Researchers started the video call by introducing themselves, developing rapport, and thanking the participants for their time. Participants were presented with two practice tasks, (including to talk aloud the thoughts that went through their minds when deciding on what pair of shoes to wear that morning) to ensure alignment with researchers’ aim of the session. After each practice task, researchers provided feedback regarding what are thought statements (e.g., “It is raining outside, so I should not wear white shoes”) and what statements are analyses of participants’ own thoughts (e.g., I decided to wear brown shoes this morning because they would not be marked by the rain”), affirming that the former is what we were looking for.

Following the practice tasks, participants were presented with a 1-minute video introducing the goals of the AI-based clinical decision support system without specifically outlining the inner workings of the machine learning algorithm nor variables displayed in the mock user interface. Care was taken to ensure that the video did not prime participants to answer any questions in a socially desirable manner. This ‘warm-up’ took approximately five minutes.

After the video, all participants were shown two static displays that were patient scenarios of varying complexity with their corresponding mock user interface (i.e. Artifact 1 and Artifact 2). Artifact 1 was shown in two stages: prediction score only, and then prediction and explanation. Participants were given a simple prompt with the prediction score of Artifact 1: “This is a prediction score that comes from the AI system. It is linked to a patient. Can you tell me what you are thinking?” and when the explanation was also displayed: “I am adding this layer of information. This information is about the same patient. Can you tell me what you are thinking?” All participants were shown the same artifacts.

After TAP, participants were asked to respond to five questions with binary response options, which were: 1) Was the AI system confusing or not confusing 2) Was the AI system overwhelming or not? 3) Was the AI system missing something or complete? 4) Was the AI system not useful or useful? and 5) Was the AI system misleading or clear?

To conclude, we asked two ecological validity questions: if participants would use the system in their current work; and if participants would recommend its use to a colleague. The protocol was designed to take approximately 20 minutes to go through. Video calls were conducted in English and digitally recorded. Where there was a language barrier, a translator was present to ensure thorough understanding of the process and participants were given the liberty to respond in Spanish.

### Materials

From a dataset of 15,337 cancer patients, we identified a cohort of 1,348 early stage non-small-cell lung cancer patients following criteria provided by medical experts to predict risk of tumor recurrence. Patients’ data included demographics features, diagnosis features, symptoms, comorbidities, smoking information, and treatment received (surgery, chemotherapy, radiotherapy)–see Table 3 [34]. Pre-processed data was then modeled as a Knowledge Graph [35], on which we trained a graph representation learning model [36, 37].

**Table 3:** Patient inclusion/exclusion criteria, reproduced from [34].

Feature Name:	Case 1	Case 2	Exclude
	Include a patient if the listed feature’s value is among one listed in this column.	Include a patient if the listed feature’s value is among one listed in this column.	Reference of which patients were excluded, because of feature’s values not being listed in Case 1 and/or Case 2.
Cancer Type	Non Small Cell Lung Cancer (NSCLC)		Small cell lung cancer, Carcinoid tumor, Epithelial thymic tumor, Mesothelyoma, Others, NA*
Tumor Stage	I, IA, IA1, IA2, IA3, IB, II, IIA, IIB	IIB	IIIA, IIIB, IIIC, Otros, IV, IVA, IVB, Limitado, Extendido, NA*
Stage T	T1a, T1b, T1c, T2a, T2b, T0, Tx, Tis	T3	T3 (except Case 2), T4, NA*
Stage N	N0, N1	N0	N2, N3, Nx, NA*
Stage M	M0		M1a, M1b, M1c, Mx, NA*
***Total number of patients per case*:**	1711	375	-
***Total number of patients (Cases 1 & 2)*:**	2086		
Chemotherapy Type In the case when a patient had chemotherapy.	Adjuvant chemotherapy, QT intravenous, Adjuvant QT-RT		Oral targeted therapy, Oral and intravenous chemotherapy, Others, Immunotherapy, Concomitant QT-RT, Sequential QT-RT, Oral chemotherapy, Neoadjuvant chemotherapy, Intravenous chemotherapy + immunotherapy, Neoadjuvant QT-RT, Hormonal, NA*
Include patients who did not undergo chemotherapy.	*Include patients without chemotherapy that fit other criteria*.		-
Radiotherapy Intention	Adjuvant		Palliative, Radical, Profilactic, Neoadjuvant, NA*
*In the case when a patient had radiotherapy*.			
Include patients who did not undergo radiotherapy.	*Include patients without radiotherapy that fit other criteria*.		-
***Full Cohort Size*:**	**1348**		

*NA—Not Available meaning there is entry about a patient, but value is either NULL or—or NA.

Ghassemi and colleagues argue that it is misguided to expect that clinicians can assess the quality of an ML output to make decisions about individual patients. In their words, “contemporary explainability techniques… can produce broad descriptions of how the AI system works in a general sense, but for individual decisions, the explanations are unreliable, or in some instances, only offer superficial levels of explanations” [38]. However, we argue that local, post-hoc explanations tend to provide simple and intuitive support to a prediction albeit at the expense of the complex mathematical model that underpins the prediction in the first place. We therefore produced two stimuli (one simple and one complex) to test our hypothesis.

In preparing the stimuli, we asked an oncologist who is not part of this study to pick two patient cases of varying complexity from the identified 1,348 patient cohort. The two patient cases, marked as test cases, were excluded from the training set. We trained a graph representation learning model for predicting the risk of tumor recurrence on the training set and calibrated it on the validation set.

We used the trained model to make predictions for these two test patients. Predictions were generated and accompanied by a post-hoc, local, example-based explanation that highlights influential patients from the training set [33].

For this study we prepared two stimuli, each presenting both prediction and explanation. Predictions were obtained from the graph representation learning model (ComplEx-N3) [39, 40]. The model achieves 68% accuracy on a 200-patient held-out test set [34]. A mock user interface rendering was developed for each of the two test patient cases. Appendix A in S1 File shows the two stimuli that were displayed to the oncologists as part of the protocol. Artifact 1 is a clinically straightforward patient, while Artifact 2 shows a more complex case. Artifact 1 was presented in two stages: only the prediction score; and then the whole display. This way, researchers could examine participants’ assessment of the prediction score alone, and then as a whole, which includes the explanation model. Artifact 2 was presented whole.

The think aloud protocol and materials (see Appendix A in S1 File) were piloted with a senior ML researcher and an independent oncologist prior to being used in the study.

### Data analysis

Midway through the completion of the video calls (n = 6), the researchers met with an independent senior researcher to go through initial thoughts and reflections. It was deemed that saturation has been reached, but that the remaining scheduled video calls (n = 4) should be conducted to get a better idea of the ecological validity of the CDSS. When all the participants have been through the protocol, audio recordings were transcribed and analyzed using inductive thematic analysis [41]. Two transcripts in Spanish were translated into English by the Project Manager of CLARIFY. Two researchers independently reviewed half of the transcripts and annotated specific words, phrases, and sections that were linked to larger ideas, themes, and sub-themes (i.e. possible ‘codes’). Next, these notes were reviewed together, and potential strategies to organize the ideas, themes, and subthemes were reviewed and standardized alongside an independent senior researcher. Using the agreed coding strategy, researchers reviewed and annotated all the transcripts together. Any discrepancies were resolved through examining the contextual evidence around the point of contention and double-checking tone and presumed intent with the researcher who delivered the protocol.

## Results

All participants were presented with the stimulus of the prototype interface and asked questions in English. Two of 10 participants preferred answering in Spanish. The audio recordings of their TAP were translated into English. TAPs took between 13 to 18 minutes to conclude. Data analysis took place the week after they were conducted.

Direct quotes are represented by “double quotation marks,” rephrased quotes are represented by ‘single quotation marks,’ and addition by the researchers are indicated by [square brackets].

The approach chosen, TAP, elicited participants’ thoughts, both positive and negative, when viewing the prediction and explanation system for the first time.

Results from the series of binary-response questions and ecological validity questions are presented alongside the themes. Through the thematic analysis, we identified five themes from the data: concept/execution gap, manifestation of process, perception of credibility, perception of utility, and frozen vs. dynamic. Below, we describe the five themes.

### 1. Concept/execution gap

This refers to the limitations of the current example-based explanation model (execution) in fully enabling the objectives of the CDSS. Participants generally understood the goals of the system and the need to integrate decision support tools in clinical practice, especially as other systems which predict other types of cancers exist, “This is a good idea because in lung cancer we don’t have anything like this, and this would benefit survival if we can treat earlier” (Participant 3).

All participants generally acknowledged the potential benefit of the system to assist in decision making, but were skeptical of the current system’s ability to deliver. Two of 10 participants specifically recalled that when presented with just the percentage relapse score, they found the prediction was clear and were not confused. However, 7 out of 10 participants stated when presented with the relapse score and explanation, they were confused by the influential examples (other patients) that served as the explanation. Participant 10 stated, “I would like someone to explain it to me more. I know this is very difficult so I won’t understand all the AI explanations…[but] I would want someone to explain the method more.” Overall, most (7 out of 10) stated they were confused or misled when the example-based explanation was displayed alongside the prediction score.

Only three participants understood the approach of example-based explanations which was based on influential patients. Participant 5 described, “I think one part is my patient, another is the analysis and then, the other case, I don’t understand what does including another case provide.” Here "the other case" or "another case" refers to the ‘influential examples’–other patients from the database whose clinical characteristics are similar to the patient in question.

Although most participants indicated they were confused by what they were seeing, only half (5 out of 10) indicated they were overwhelmed by the system. Participant 5 stated, “As a clinician, what [is] important to me is the risk of relapse, that’s what I’m going to use…the rest provides extra information that we are not all going to use.”

### 2. Manifestation of process

This refers to the explanation system being perceived as indicating and showing the mathematical processes utilized by an ML system to achieve its desired purpose, and the extent to which that process is perceived by the participant.

In the present study, example-based explanations were deemed by the developers as sufficient in showing the processes of the ML model through the representation of patients whose clinical features are closely related to the patient in question. While both Artifacts 1 and 2 indicate on the mock display that the prediction score was based on a sample of over 1,000 lung cancer patients, it was not intuitive to our participants that the relapse score was based on the entire database of patients, as only two or three ‘influential examples’ were displayed.

“I think the relapse score is important information, but was this patient compared with the 2 other patients here or a bigger group of patients? I think it’s very difficult to do a prediction for relapse only by comparing with a small sample. The idea is very good but it is necessary for us to know the original information from the total group of patients.”–Participant 6

In showing the ‘influential examples’, some participants assumed that the system regurgitated information from the electronic medical record system, without adding additional layers of analysis: in other words, the machine learning effort was obscured.

“The table gives information about the patient that I already know. This is information I give to this system. But I want to know why, which are the high-risks [variables].”–Participant 3

### 3. Perception of credibility

This refers to the oncologists’ mental representation of how convincing or believable the outputs of the AI system are. The first display which the participants saw was a prediction of relapse score without any explanation. The prediction score alone, without the explanation, was not sufficient to most participants. As Participant 3 said, “only knowing the 61% is not enough.” As expected, participants did not trust the prediction score alone without the explanation. Indeed, the predictions and explanations did not include any description of the accuracy of the ML model in a way which medical doctors would be used to, e.g., confidence intervals or p-value.

The added visual of example-based explanations helped participants better understand that the prediction score is supported by data about the patient and other patients. This is in comparison to when the percentage risk score alone was displayed (with the example-based explanations hidden from view).

“Here everything is clearer, there’s more data about the patients. It expresses pretty well the differences between them and what they have in common. It seems pretty clear, it’s quite visual. It seems simple.”–Participant 4

To some participants, example-based explanations did not adequately explain the prediction score. They questioned the prediction score for the complex case as the ‘influential example’ seems to have very different clinical features.

“This is not correct for me. We can’t compare these patients because the type of treatment and type of surgery is different. The prediction [should be] very different for both patients.”–Participant 6

Participants highlighted the lack of order of priority in patient attributes displayed in the explanation. The important attributes contributing to the relapse score was not displayed in an intuitive way.

“The descriptive part of the different variables are confusing. Because obviously there are some that are really important, but others could be too general. For example, the comorbidity, cardiopathy is just like saying nothing because it could be [a] very important issue or it could be arrythmia, well controlled, with no implications. In the same category, dyslipidemia could mean nothing in the evolution of the patient. It depends. Hypertension could be very difficult to control. I think it is quite general. Also, a smoker, how much? I don’t know, it is too much information and too general to modify my individual decision over a patient.”–Participant 1

Among the five participants who did not find the explanation overwhelming, three participants struggled to understand the value of having so much information displayed. Participant 1 explained, “There’s a lot of information that is not really important, because I don’t care if you show me [other patients] … I’m seeing patients similar to the one I should decide [about] is what I think [the display is showing]. But looking to the attributes of 2 similar patients is not important. The most important is the conclusion of the study from the overall population, not just two.”

### 4. Perception of utility

This is the oncologists’ mental representation of how valuable the system is in helping them achieve specific care goals. The objective of the system is to assist in adjusting therapeutic guidelines for lung cancer and inform attending physicians of when their patients should be asked to return for a follow-up. Participants noted that the prediction score is clear.

“Well, first the information about the risk of relapse of 61% it’s important. … I think the information is clear. I think it’s the most important.”–Participant 5

Despite the clarity, participants found that the system overall “is too general to take out a conclusion” and does not help in deciding “what treatment I would provide.” The example-based explanations were insufficient to feed into a decision-making process.

“If we had a platform with a lot of information, we would use it no doubt. I think it is a good idea and is really necessary.”–Participant 3

Some (4 out of 10) of the participants believed that the system was missing something and was not complete in the current form. Participant 2 stated, “[The system] is missing a lot of things. I need to know why. What is the most important attribute to [indicate] relapse?” Overall, some (4 out of 10) found the system to be useful in its current form.

Interestingly, some participants thought the current system would be more helpful for research instead of for use in daily clinical work.

“This is more useful for research or for comparing patients but in our daily work it doesn’t provide much information. It provides information to compare patients, it’s more general, not for individual patients. To compare one, two or three patients it’s not very relevant clinically speaking. The example is quite clear but it’s not very relevant in our daily work, to tell you the truth.”–Participant 4

Many (6 out of 10) participants stated they could not see the system fitting in to their working day. Among those six, four participants said they would use it if there were changes made to it. Further, only a few (3 out of 10) stated they would recommend it to a colleague but others would be more likely to recommend if there were changes or if they had the opportunity to use it more. Participant 3 explained, “The idea is really useful. By improving everything, the platform could be very useful. Now it is not useful enough.”

### 5. Frozen vs. dynamic

This refers to the machine learning algorithm providing a static prediction score, based on patient’s clinical data that was entered into the system. A frozen system is static, in contrast to a dynamic system which provides real-time updates to patients’ risk scores if hypothetical care scenarios or patient characteristics changed.

“It would be more useful if we have a calculator. That I could put for example, their age, tumor, their stage into a calculator and then have the relapse prediction.”–Participant 9

Participant 3 noted, “I want to know if I give treatment early, does this improve survival?” Several participants suggested that a dynamic prediction score, with information on how the relapse prediction could be reduced by therapy, would be more useful.

“I can see me using this but with more information. I need to know if you put a kind of chemotherapy, something will happen. If you use this type of therapy, you will have better outcomes. [We] need to know something more.”–Participant 2

Some participants further noted that a treatment-oriented system, with projected relapse scores based on various therapeutic options, would be preferable. An existing predictor system with this feature has been used by the oncologists before.

“For example with the Breast Predict, it’s a breast cancer predictor. I would go for something like that, not comparing with 1 or 2 patients but with a huge database of lung cancer patients where you insert the data and you have the risk of relapse according to the different treatments.”–Participant 4

## Discussion

This study describes the use of TAP with clinicians to help developers assess the interpretability of an explainable AI system for lung cancer relapse. It could elicit clinicians’ thoughts on the pros and cons of the predictive system and its example-based explanations. Using TAP, participants were not given any ‘leading’ prompts and questions; the neutrality of the protocol enabled a free expression of thoughts. We found that TAP was able to elicit information that is more useful than several months of desiderata gathering process with the clinicians. The results confirm our expectation that perceptions of credibility and utility acted as a proxy for quality of the explainable AI system in the absence of recognized quality standards.

Most participants noted that while clear, a prediction score alone was not sufficient to help them trust the system. Showing the example-based explanations helped some participants with their *perception of credibility*–that some machine learning process took place–although participants were no less confused by the information displayed. Further, participants expressed that the prediction score alone would be useful in prompting them to alter their care plan for patients with high relapse prediction. However, the explanations were found to have mixed important and less important information which could obscure the value of the predictive model for informing or altering care plans (*perception of utility*). From the TAPs, we gathered that that this was influenced by a *concept/execution gap*–the limitations of the current explanation system (execution) in fully enabling the objectives of the system. While clinicians were favorable towards the concept, they were confused by the example-based explanation model, believing that the prediction score were influenced by 2–4 influential examples only, rather than the >1,000 cases that make up the training set. In this regard, a stronger *manifestation of process* was required, which could be a different explanation model or a better user interface, but designed with the goal of influencing the perception of credibility. As the predictive model was presented as a probability which relies on a point estimate, future attempts could include uncertainty quantification. Lastly, participants seemed to have expected a dynamic system with changing relapse prediction score depending on hypothetical care scenarios, rather than one that is *frozen* or static. Participants’ expectations of what-if analyses are common in financial and meteorological forecasting, and future attempts at developing explainable AI systems could combine these with uncertainty quantification to truly assist clinicians in developing a robust treatment plan.

Our findings show that the feeble *manifestation of process* negatively influenced the perception of credibility. Credibility, being a subjective appraisal, is very difficult to measure. Is credibility a question of pedagogy? Arguably, medicine is not an exact science [42]. This manifests in variability in clinical decision making, and contradictory second or third opinions. Diagnostic criteria tend to have arbitrary cut-offs, but as the brain is wired to function quickly using heuristics, few clinicians would question what they’ve come to believe as truth. It has, perhaps, become important for clinicians to see a specific type of *manifestation of process*: where the inner workings of the ML system are shown to be aligned with their own heuristics (confirmation bias). Our findings show that those who found the explanations overwhelming still said the system was missing something.

TAP lifted the curtain for the previously unspoken or unknown barriers between developers and expert-users. In previous attempts to determine user requirements, features that were determined essential by this study’s participants were never mentioned. For example, requirements of specific ways of indicating the accuracy of the predictive model were never formalized. Our findings show that different methods for gathering desiderata in the ML development stage will be advantageous, using methods that consider the expertise and academic background of the end users as well as the context of the use [17]. TAP places the power solely on the experts while those delivering the protocol remained largely passive. While simple, this method provides high returns in the form of expert-users’ unbridled assessments of a system which they are meant to use in their daily work. In the development of high stakes systems, such as one that predicts lung cancer relapse, to elicit expert-users’ needs is of utmost importance. This key step enables the developers of the system to alter the explanation system and further refine the features highlighted by the explanation.

There are two kinds of implications: first, relating to the value of this study for the CLARIFY project, and second, relating to XAI development in general.

TAP helped the developers of the CLARIFY system to better understand the expectations of the oncologists. By eliciting their stream of thought when presented with the artifacts, it became clear what features oncologists found useful and challenging. The protocol enabled the developers to listen to various voices on missing features and expectations—elements of the system that the oncologists deemed important to trust the system, for example, clearly conveying information on the cohort size, selection criteria, and model accuracy. From the results of the study, the developers noted that it is crucial to include such context in the explanation model. Add on questions to the TAP allowed the developers to closely follow participants’ assessment of the prototype as well as specific features they disliked and would have liked to see instead. These are details which participants did not express in previous sessions aimed at gathering desired features early in the development process, including features to be presented as part of context-setting and the way relevant information should be presented by the explanation system. One notable insight here was the potential for example-based explanations to cause confusion.

Second, there is a set of implications for machine learning in the context of CDSS. System development in general should consider how clarifying expectations for the ML system and accompanying explanations on an early prototype save time and costs which accrue from misalignments, especially in multidisciplinary teams with different areas of expertise, expectations, and goals. As expected, TAP was shown to be able to guide system design much better than a series of unstructured requirements gathering sessions that the developers had with the oncologists earlier in the project. The latter is proposed to work better with final design features such as fine tuning the user interface. Explanations do not yet come in a standard, agreed-upon form, and are hard to get across to expert-users, who are searching for familiar means of information delivery. Future research should consider forming a standard form of explanations acceptable to various professions. In short, TAP could be a low-cost way for ML developers to understand expert-users’ thought processes as they come across explainable AI systems that they must integrate into their judgement and decision-making processes. This is arguably generalizable to other fields of (non-ML) expertise.

### Limitations

There are several potential limitations of our study that must be acknowledged. First, the potential for contamination as all participants work in the same hospital, in the same department, and have regular meetings. Second, the nature of TAP is such that is likely unable to capture the complete thought process especially when exposure to the artifacts present a high cognitive load. Research into ‘introspection’ found that people might not be able to accurately report on their own cognition, and that attempting to do so might not be underpinned by true introspection [43]. Third, TAPs were conducted in English, with participants whose primary medium of communication is Spanish. It is possible that communicating verbally in a non-native language limits the complete and full expression of what expert users are thinking, in an already unusual experience for the participants. The concurrent cognitive loads of reading through the interface and verbalizing thoughts may well have had different effects on individual participants. Thinking aloud in a different language makes it even more complicated. Furthermore, it is questionable whether clinicians could provide details on what features are desirable in an explanation system, as ‘you don’t know what you don’t know.’ On a more practical level, think aloud is dependent on how comfortable the participants are with verbalizing their thoughts. Sparse data, therefore, could be difficult to analyze and synthesize into meaningful findings.

## Conclusion

This study demonstrates the practical utility of adopting TAP to probe clinicians’ reasoning about the outputs of a prototype clinical decision support system (CDSS). The psychological investment of clinicians in their work means that ML developers need to tread carefully when building CDSS to be used in clinical contexts. There is as much judgment as science in interpreting the outputs from TAP, and researchers and ML developers alike should note that using TAP in their work requires practice and iterative efforts in order to maximize its positive impact on the eventual CDSS. Understanding where and when TAP fits in an overall explainable AI development process will require further research.

## Supporting information

S1 File Appendix A: Think aloud protocol. (DOCX)
