# Supplementary material for: Examining explainable clinical decision support systems with think aloud protocols
Source: PLoS One. 2023 Sep 14;18(9):e0291443. doi: 10.1371/journal.pone.0291443 (PMC10501571; doi:10.1371/journal.pone.0291443)
Supplement: S1 File — (DOCX) [file pone.0291443.s001.docx]

# Appendix A: Think Aloud Protocol

**CLARIFY Think Aloud Protocol**

- Interviews will be scheduled for 60 minutes.
- Tasks should take around 45 minutes to complete.
- Interviewers must introduce themselves, their roles during the interview.
- Lead Interviewer must go through the information leaflet, check for clarity and understanding, and perform the consent procedure for recording and use of quotes in subsequent reports or publications.
- Iterative approach: THIS PROTOCOL IS TO BE REVISED AFTER THE FIRST 2 INTERVIEWS.

**Introduction to Think Aloud**

During this interview, you will be asked to perform a series of tasks. I would like you to verbalise everything that you usually say to yourself silently. Please share with me your thoughts as they come. It could be anything, please do not be embarrassed. There is no right or wrong answer.

WE ARE NOT HERE TO ANSWER YOUR QUESTIONS. Any questions are in fact data.

We will go through two warm-up tasks. I will give you feedback after every warm-up task to clarify what I am looking for.

**SAMPLE TASK 1**

Please tell me how you chose which pair of shoes to wear today.

Sample prompts*:*

*Differentiate thoughts from analysis of own thoughts.*

*Thoughts are what I am looking for.*

*Keep talking to me.*

*Keep thinking aloud.*

Provide feedback

**SAMPLE TASK 2**

I am now going to share my screen, to show you an image.

*Show the image below.*


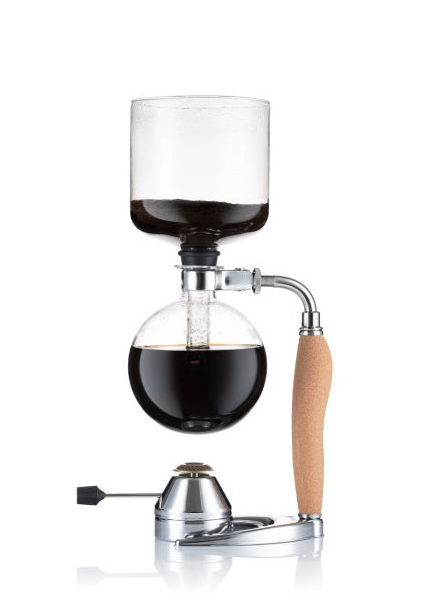


Please tell me your thoughts.

Sample prompts*:*

*Differentiate thoughts from analysis of own thoughts.*

*Thoughts are what I am looking for.*

*Keep talking to me.*

*Keep thinking aloud.*

Provide feedback

Now we are moving on to the next part of the interview. I am going to show you a series of images. These images show the information you can find on the AI system which will be introduced in your Oncology department in the near future.

(Introduction to the AI System)

**Keep in mind how you usually define the time to review or follow-up with your patients, and how you can design future treatments that are not currently in the guidelines, based on some clinical information about the patient’s relapse or progression.**

I would like you to keep verbalising everything you usually say to yourself silently. Again, there is no right or wrong answer. I am interested in hearing what you think.

**TASK 1**

*Artifact 1A: Prediction score without explanation.*

This is a prediction score that comes from the AI system. It is linked to a patient. Can you tell me what you are thinking?


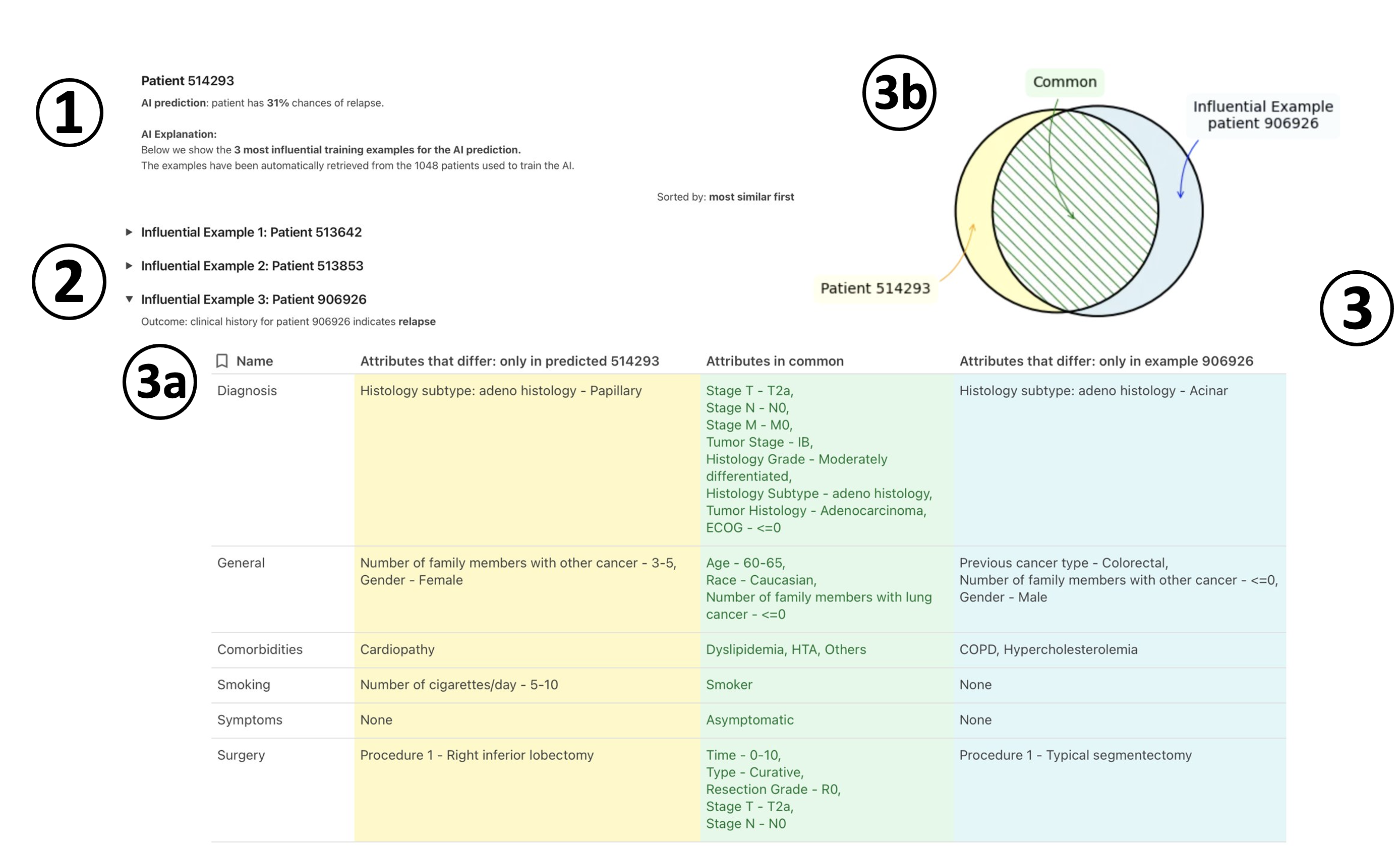


*Artifact 1B: Prediction score* ***with*** *its explanation added (simple case).*

I am adding this layer of information. This information is about the same patient. Can you tell me what you are thinking?

Now we are going to move on to the second part.


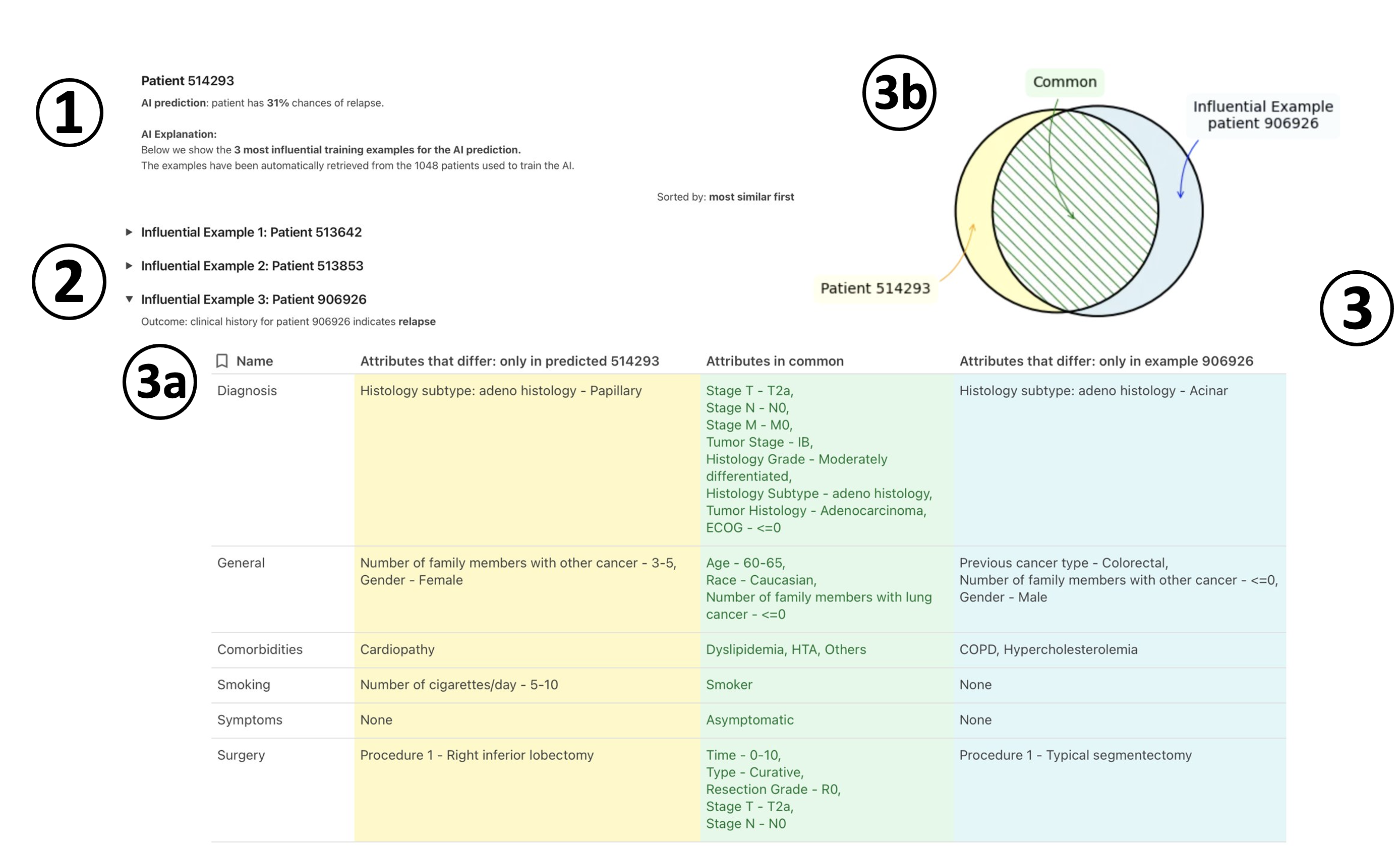


**TASK 2**

*Artifact 2: Prediction score with explanation (complex case).*

This is a different patient. Can you tell me what you are thinking?


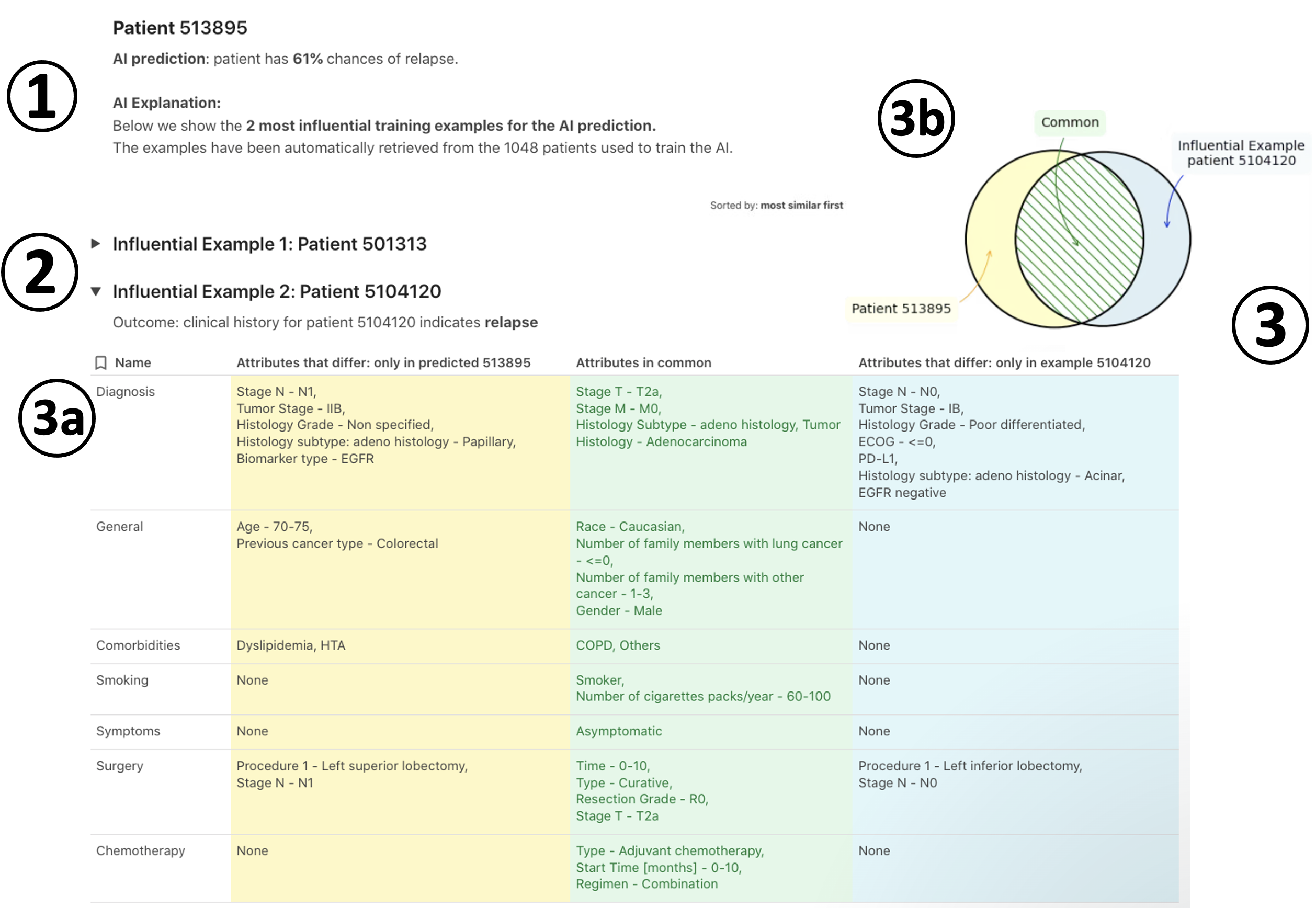


**We are done with the interview now.**

I have some last questions for you.

Was the system confusing or not confusing?

Was the system overwhelming or not?

Was the system missing something or complete?

Was the system useful or not useful?

Was the system misleading or clear?

Can you see yourself using the system in your work?

Would you recommend the use of this system to your colleagues?
